# Integrating pathological morphologies and molecular profiles for clinical biomarker discovery with unified multimodal embedding

**DOI:** 10.1002/ctm2.70765

**Published:** 2026-08-05

**Authors:** Chaofei Gao, Qian Zhang, Peng Zhang, Shao Li

**Affiliations:** ^1^ BNRist/Department of Automation Bioinformatics Division Institute of TCM‐X/MOE Key Laboratory of Bioinformatics Tsinghua University Beijing China; ^2^ Department of Formula and Syndrome Research, Institute of Basic Theory for Chinese Medicine China Academy of Chinese Medical Sciences Beijing China

Spatial omics technologies have revolutionised our understanding of tissue architecture, revealing novel molecular biomarkers for clinical diagnosis and disease management.^1^ Despite these advances, the clinical deployment of spatial omics remains limited by high sequencing costs and complex experimental workflows.^2^ Moreover, biomarkers discovered in this manner are typically validated only in small cohorts, lacking the generalizability required for broader clinical populations.^3^ By contrast, cost‐effective and widely available haematoxylin and eosin (H&E)‐stained pathology slides have remained the gold standard in oncology for decades.^4^ However, H&E slides capture only morphologies, lacking the detailed molecular information. Thus, integrative analysis of pathological morphologies and molecular profiles holds the key to translating spatial biology into precision oncology, enabling the discovery of generalizable biomarkers for clinical application.

However, current multimodal methods integrating morphologies and molecular profiles typically fall into a dilemma between interpretability and generalizability.^5^ On one hand, top‐down approaches start from clinical phenotypes and employ fully supervised models to predict outcomes, achieving high accuracy but offering limited biological interpretability.^6^ On the other hand, bottom‐up approaches start from defined molecular mechanisms to identify biomarkers anchored to specific biological processes, yet these biomarkers often exhibit poor cross‐cohort generalizability, hindering their clinical applications.^3^

Bridging this gap requires a multimodal framework that accurately characterises clinical outcomes while revealing the cross‐modal associations that underpin them. Here, we introduce Multi‐Embed,^7^ a recently published self‐supervised framework enabling synchronous cross‐modal inference and integration of multilevel pathological morphologies and multilayer molecular profiles with unified embedding. Based on these capabilities, Multi‐Embed enables clinical biomarker discovery with both generalizability and interpretability across multiple scenarios, offering a practical route to bridge biological discovery with clinical practice.

## 1 METHODOLOGICAL OVERVIEW

**FIGURE 1:**
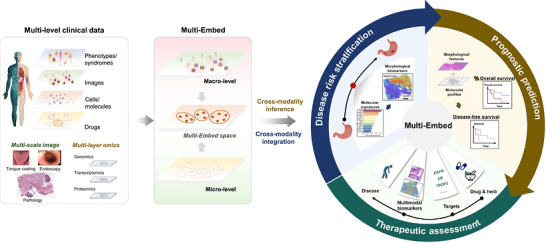
The paradigm of clinical biomarker discovery using Multi‐Embed.

Multi‐Embed is established upon the principle of cross‐modality network modular association, which was identified through systematic analysis of multi‐level data spanning traditional Chinese and Western medicine (Figure 1). It establishes a unified embedding space that bridges macroscopic morphologies and microscopic molecular profiles, enabling cross‐modality inference and integration.

The advantages of Multi‐Embed lie in its capacity for both deeper mechanistic insight and broader biological coverage. In terms of depth, it achieves state‐of‐the‐art performance in cross‐modality integration with substantially higher predictive accuracy than existing methods, while maintaining biological interpretability. This reveals the associative and complementary patterns between morphological and molecular features in the unified embedding space. As for breadth, trained on multimodal data across 12 cancer types, it demonstrates generalizability across both cohorts and scales, accommodating pathological morphologies from slide and spot to single‐cell resolution and molecular profiles from genomics and transcriptomics to proteomics.

## 2 GENERALIZABLE AND INTERPRETABLE BIOMARKER DISCOVERY

Building on its methodological advances, Multi‐Embed provides a practical route to biomarker discovery with both cross‐cohort generalizability and biological interpretability (Figure 1). Through cross‐modality inference, it enables bidirectional translation between molecular signatures and morphological features, predicting molecular biomarkers from pathology slides or identifying morphological indicators of known molecular markers, an approach particularly valuable when molecular profiling is unavailable. Through cross‐modality integration, it facilitates the discovery of multimodal prognostic biomarkers that achieve higher predictive accuracy while retaining mechanistic interpretability. Together, these capabilities position Multi‐Embed for clinical biomarker discovery across the disease trajectory, from risk stratification and prognostic prediction to therapeutic assessment.

In terms of disease risk stratification, Multi‐Embed leverages cross‐modality inference to translate established molecular risk signatures into the corresponding morphological features, thereby deriving clinical biomarkers grounded in molecular mechanisms. This approach is essential as disease onset is a prolonged, low‐probability process that requires longitudinal monitoring across large cohorts, making large‐scale molecular profiling impractical due to cost and complexity. By learning cross‐modality associations from limited but high‐quality multimodal data, Multi‐Embed enables de novo discovery of morphological biomarkers through feature retrieval based on the molecular signatures, enabling pre‐disease risk stratification on a large‐scale sequential cohort. A representative application is the identification of biomarkers for extremely early gastric cancer (EEGC),^8^ a premalignant state indicating tumorigenesis, which was molecularly defined by a previous study yet lacking standardised diagnostic criteria in routine histopathology. Morphological biomarkers associated with EEGC were identified using Multi‐Embed and subsequently validated in a large‐scale sequential cohort for their ability to distinguish progressive from regressive lesions.

In the disease phase, cross‐modality integration enables Multi‐Embed to discover the multimodal prognostic biomarkers. Using attention‐based fusion, Multi‐Embed predicts both overall and disease‐free survival with accuracy surpassing existing approaches.^6^ Beyond prediction, it simultaneously captures the spatial organisations of cells and molecular signatures related to the clinical outcomes, thereby identifying the multimodal biomarkers. This integrative approach was validated across 12 cancer types, where it stratified patients into two distinct prognostic groups: one enriched for tertiary lymphoid structures corresponding to favourable outcomes, and another marked by epithelial‐mesenchymal transition associated with poor prognosis. Together, these findings illustrate a biomarker discovery paradigm that couples biological interpretability with cross‐cohort generalizability.

For the treatment stage, Multi‐Embed draws on both its inference and integration capabilities to advance therapeutic biomarker discovery and drug target identification. On one hand, cross‐modality inference enables the translation of known molecular targets or established drug‐response signatures into morphological biomarkers, allowing treatment response prediction without repeated molecular profiling. On the other hand, cross‐modality integration uncovers de novo multimodal biomarkers of treatment response by jointly modelling the relationships between multimodal features and clinical outcomes, thereby revealing candidate targets and drug resistance mechanisms. When Multi‐Embed was applied in a breast cancer cohort treated with trastuzumab, multimodal biomarkers identified through the above paradigm predicted treatment response with significantly higher accuracy than established molecular markers or morphological features alone, illustrating the potential of integrative approaches to refine therapeutic decision‐making.

## 3 CONCLUSIONS AND PERSPECTIVES

Multi‐Embed serves as a versatile framework for biomarker discovery that bridges basic biological insights and clinical application, enabling de novo identification of interpretable and generalizable biomarkers across risk stratification, prognostic prediction, and therapeutic assessment.

Looking ahead, the analytical framework of Multi‐Embed can be extended beyond pathology to a broader spectrum of medical imaging, including computed tomography, magnetic resonance imaging and traditional Chinese medicine modalities such as tongue coating and retinal photographs. This expansion would unlock non‐invasive screening scenarios, further amplifying the clinical utility of Multi‐Embed‐based biomarker discovery.

Furthermore, as a computational approach that systematically reveals the associations between morphological and molecular profiles, Multi‐Embed offers an extension to network pharmacology.^9^, ^10^ By integrating image data into network‐based drug discovery, it opens avenues for identifying novel therapeutic targets alongside their morphological patterns, thereby enriching the methodological toolkit for network pharmacology.

In summary, Multi‐Embed provides a holistic and systematic approach to decoding multilevel biological associations across macroscopic, mesoscopic, and microscopic information, achieving both mechanistic depth and application breadth that together advance clinical biomarker discovery.

## AUTHOR CONTRIBUTIONS

Chaofei Gao and Qian Zhang drafted the manuscript. Chaofei Gao drew the figure. Peng Zhang and Shao Li provided valuable disscussion and revised manuscript. All authors have read and approved the article.

## ETHICS STATEMENT

The authors have nothing to report.
